# Contrasted patterns of selective pressure in three recent paralogous gene pairs in the *Medicago* genus (L.)

**DOI:** 10.1186/1471-2148-12-195

**Published:** 2012-10-01

**Authors:** Joan Ho-Huu, Joëlle Ronfort, Stéphane De Mita, Thomas Bataillon, Isabelle Hochu, Audrey Weber, Nathalie Chantret

**Affiliations:** 1 INRA - Institut National de la Recherche Agronomique, UMR AGAP, Montpellier, 34060, France; 2 INRA - Institut National de la Recherche Agronomique, UMR IAM, Nancy, France; 3 Bioinformatics Research Center (BiRC), Aarhus University, Aarhus, Denmark

**Keywords:** Duplication, Medicago, Neofunctionalization, Subfunctionalization, Paralogs evolution

## Abstract

**Background:**

Gene duplications are a molecular mechanism potentially mediating generation of functional novelty. However, the probabilities of maintenance and functional divergence of duplicated genes are shaped by selective pressures acting on gene copies immediately after the duplication event. The ratio of non-synonymous to synonymous substitution rates in protein-coding sequences provides a means to investigate selective pressures based on genic sequences. Three molecular signatures can reveal early stages of functional divergence between gene copies: change in the level of purifying selection between paralogous genes, occurrence of positive selection, and transient relaxed purifying selection following gene duplication. We studied three pairs of genes that are known to be involved in an interaction with symbiotic bacteria and were recently duplicated in the history of the *Medicago* genus (Fabaceae). We sequenced two pairs of polygalacturonase genes (*Pg11*-*Pg3* and *Pg11a*-*Pg11c*) and one pair of auxine transporter-like genes (*Lax2*-*Lax4*) in 17 species belonging to the *Medicago* genus, and sought for molecular signatures of differentiation between copies.

**Results:**

Selective histories revealed by these three signatures of molecular differentiation were found to be markedly different between each pair of paralogs. We found sites under positive selection in the *Pg11* paralogs while *Pg3* has mainly evolved under purifying selection. The most recent paralogs examined *Pg11a* and *Pg11c,* are both undergoing positive selection and might be acquiring new functions. *Lax2* and *Lax4* paralogs are both under strong purifying selection, but still underwent a temporary relaxation of purifying selection immediately after duplication.

**Conclusions:**

This study illustrates the variety of selective pressures undergone by duplicated genes and the effect of age of the duplication. We found that relaxation of selective constraints immediately after duplication might promote adaptive divergence.

## Background

Gene duplications have long been hypothesized to be drivers of genome and gene function evolution [1]. Recently, availability of large-scale sequence data, and especially entire genome sequences, has brought significant support to this view [2,3]. In plants, duplications appear to be frequent and most lineages studied up to now have been affected by whole-genome duplication events (polyploidy) and/or segmental duplications [4-10].

Starting with Ohno, a range of models has been proposed to predict the fates of paralogous gene pairs resulting from duplications. These models can be categorized by their assumptions: they can be either neutral or involving natural selection, and can consider the early stage of duplication, *i.e.* when the duplication is not yet fixed in the species or start with the assumption that the gene duplication has just been fixed (recently reviewed in [11]).

Immediately after the gene duplication event, the two copies are assumed to be identical and therefore functionally redundant. At this stage, there should be no selective pressure against any loss-of-function mutation affecting either copy. As a result, it is believed that most instances of gene duplications will eventually result in the loss of one of the copies (pseudogenization or nonfunctionalization). However, the relaxation of purifying selection (due to the initial redundancy) may allow some amount of divergence and occasionally can let one copy acquire a new function and be subsequently maintained by natural selection (neofunctionalization). This scenario is essential for the creative role of duplication envisioned by Ohno [1]. Force *et al.*[12] suggested that the presence of two redundant genes may drive the fixation of complementary degenerative mutations in both of copies, with higher probability in gene regulatory regions. At the end of this process, both gene copies are required to perform the set of functions originally performed by a single gene (subfunctionalization). These two scenarios are not mutually exclusive and may act jointly [13]. Besides these models, the maintenance of functionally redundant copies (without functional divergence) could be adaptive under specific circumstances, either through dosage effect or as a means of genetic robustness against deleterious mutations [14-16] and therefore also explain the fixation of duplications in species [11].

Functional analyses have been performed in order to determine the relative importance or the interaction between these different models. The occurrence and the characteristics of functional divergence of paralogous genes can be addressed either through the regulatory or protein-coding sequence angle.

Whole-genome expression profiles revealed divergent expression patterns between paralogous gene pairs, providing indirect evidence for subfonctionalization and/or neofunctionalization [17]. Similar conclusions were also drawn from studies of polyploid species for which duplicated genes were instantly fixed in the species founder individual [18-20]. More specific and detailed functional analyses revealed several cases of paralogs undergoing neofunctionalization or subfunctionalization [21,22].

Beside differences in gene expression, rates of molecular evolution can be used to qualify the constraints experienced by genes. In particular, contrasting the rate of protein-changing (non-synonymous) substitution (dN) and the rate of silent (synonymous) substitution (dS) at the nucleotide level allows qualifying the type of selection acting on individual gene copies after a duplication event. The intensity of purifying selection is often estimated through the ratio ω = dN/dS. Values of ω < 1 are interpreted as evidence for purifying selection (the lower ω, the stronger purifying selection). Following pseudogenization, ω = 1 is expected (no constraint). Last, amino acid sites exhibiting ω > 1 are likely directly targeted by positive selection. As an example, the evolutionary fate of ten genes recently duplicated by retrotransposition in mice was studied by contrasting synonymous and non-synonymous rates [23]. Gene duplications have been the subject of many functional and molecular studies in plants [24,25], but here we aimed at analysing specifically the selective constraints exerted on duplicated genes through analysis of their rates of substitution. In order to shed light to the temporal variation of selective constraints acting on duplicated genes following their duplication, we focused on the evolution of fairly recent duplicated genes at a time scale appropriate for coding sequence evolution rates analysis. Such study can provide insight about the relative role of relaxation of purifying selection and positive selection in the fate of duplicated genes.

We investigated rates of molecular evolution of three duplicated gene pairs in the genus *Medicago* (L.), therefore maximizing the amount of available phylogenetic signal. We selected gene pairs involved directly or indirectly in the symbiotic interaction between legumes and nitrogen-fixing bacteria (rhizobia). The first genes code for polygalacturonases, which are enzymes involved in the degradation of polysaccharides. One member (*Pg11*) is involved in pollen tube elongation and the other (*Pg3*) in the tip growth of the infection threads during the establishment of the symbiosis with nitrogen-fixing bacteria *Sinorhizobium* sp [26]. The second genes are *Lax* (Like-*Aux1*). They are auxin efflux carriers and play an important role in auxin-controlled processes such as tissue growth and in particular development of nodules.

Mutualistic host-symbiont interactions present the interest of combining several features we can expect will promot fast evolution. Mutualisms are often based on nutrient exchanges and involve strong selective pressures, since both costs and benefits are important. The interaction with a biotic partner can cause shifting selective optima, especially if there are conflicts of interest. Finally, in contrast with host-pathogen interactions, mutualisms can involve the evolution of novel structures by both partners. The legume-rhizobium symbiosis evolved relatively recently, around 60 million years ago, culminating with the emergence of a specific organ, the root nodule [27]. Therefore, the genes underlying rhizobial symbiosis in legumes are likely to record the signatures of past selective pressures caused by the emergence and diversification of symbiosis as well as pressures linked to their current function. Due to a whole-genome duplication event that occurred approximately 58 Myr ago [28], legumes are therefore a good model to examine the changes of selective pressures over time for duplicated genes.

Rates of molecular evolution of paralogous gene copies (hereafter paralogs) should be studied preferably in a variety of species to have enough power to inner substitution rates. Moreover paralogs should be characterized in a set of extant species that have diverged after the ancestral gene duplication. In spite the growing availability of full genome sequences, plant model species are usually not related enough to allow for analysis of divergence at the nucleotide level. In the case of the Fabaceae family, three species have been sequence (*Medicago truncatula*, *Lotus japonicus* and *Glycine max*), but their divergence times would represent a time scale of 50–60 million years [29]. Moreover, more taxa are needed for contrasting early and late selective pressures. We re-sequenced three pairs of relatively recently duplicated genes in 16 other species of the *Medicago* genus (in addition to *M. truncatula*). We chose duplicated genes that (i) are recent enough so that the signatures of evolution post-duplication are still detectable, (ii) predate the speciation events within the *Medicago* genus, so that each copy is found within all species and (iii) contain at least one gene demonstrated or strongly suspected to be involved in the legume-specific symbiotic interaction with nitrogen-fixing rhizobium bacteria.

## Results

### Sequencing *Pg11a*, *Pg11c*, *Lax2* and *Lax4*

Depending of the gene, a successful amplification was obtained for a total of 10 to 17 species. The resulting sequence alignments had a length of 729 bp for *Pg* genes and 798 bp for *Lax* genes. We excluded sequences that did not encode a complete protein (due to frame shift or nonsense mutations) because they might represent pseudogenes and affect our estimates of rates of molecular evolution in functional paralogs. Accession numbers of sequences deposited in GenBank are from JN635641 to JN635687. Already available sequences GenBank accession numbers are AJ620946, AY115843 and AY115844 (for *M. truncatula* genes *Pg3*, *Lax2* and *Lax4* respectively), HQ737838, HQ736585 and HQ736701 (for *M. tornata* genes *Pg3*, *Lax2* and *Lax4* respectively). Details about the sequences obtained as well as GenBank accession numbers are given in Additional file 1.

### Phylogeny of *Pg* and *Lax* genes

**Figure 1:**
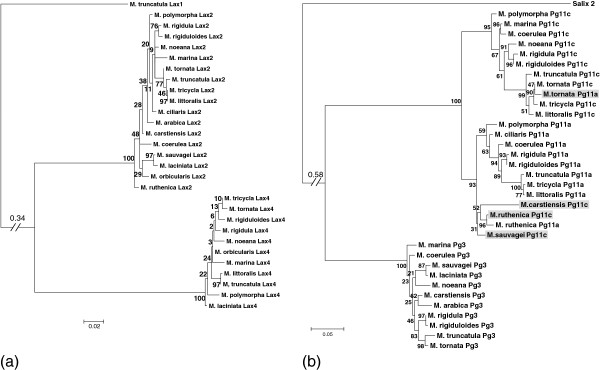
**Phylogenetic trees for *Lax *and *Pg *genes.** Phylogenetic trees obtained for *Lax2* and *Lax4* (**a**) and for *Pg3*, *Pg11a* and *Pg11c* genes (**b**). Bootstrap values (% of 100 re-sampled data set) are indicated for each branch. For presentation convenience, the branch leading to the outgroup was shortened (as indicated by an inclined double segment). Sequences underlined in light grey represent instances were a paralog is placed in the wrong clade when considering the copy specific primers used for its amplification.

The phylogeny of *Lax* and *Pg* paralogs were reconstructed using maximum likelihood and are presented in Figure 1a and Figure 1b respectively. The topologies obtained for the three paralog pairs *Lax2*/*Lax4*, *Pg3*/*Pg11* and *Pg11a*/*Pg11c* confirmed the occurrence of three duplication events predating the divergence between the 17 species we included from the *Medicago* genus. Moreover, the branches leading to each paralog clade containing the sequences of the same gene amplified from different species are well supported. Bootstrap values for the branches leading to the *Lax2*, *Lax4*, *Pg3* and *Pg11* clades are equal to 100. Within the *Pg11* clade, bootstrap values obtained for the branches leading to the *Pg11a* and *Pg11c* clades are 95 and 93 respectively. However, within the *Pg11a* and *Pg11c* clades, several inconsistencies were observed in the phylogenies: sequences obtained with the *Pg11c* copy specific primers for *M. carstiensis*, *M. ruthenica* and *M sauvagei* were placed in the *Pg11a* clade, and conversely, sequences obtained with the *Pg11a* copy specific primers for *M. tornata* were placed in the *Pg11c* clade (highlighted in grey in Figure 1b). There are several explanations for such inconsistencies: erroneous amplification (for example chimeric amplification), the amplification of a third copy resulting from an independent duplication, or genic conversion between paralogs. In order to avoid erroneous interpretations, we did not consider these four sequences further in our analysis.

The species phylogeny deduced from the data was not completely congruent between different paralogs and with the species phylogeny described in the literature [30,31]. However, within clades regrouping sequences of a same gene in the different species, branches are not well supported (Figure 1), indicating a poor phylogenetic resolution. Only three groups of species were grouped with high support, irrespective of the gene analysed. The first includes *M. tornata*, *M. truncatula*, *M. tricycla* and *M. littoralis*, the second *M. rigidula* and *M. rigiduloïdes* and the third *M. sauvagei* and *M. laciniata*. The *Medicago* genus evolved through a large number of speciation events in a short time span, and as a result, the resolution of phylogenetic relationships between *Medicago* species is difficult. Furthermore, incongruences may be observed between gene and species trees due to incomplete lineage sorting [32]. For each paralog set, we used the best fitting phylogenetic tree. We repeated the analyses of selective constraints for each gene pair using either the best topology found for the genes considered or a tree topology from the literature [30]. Results were very similar and conclusions were not affected. Consequently, only results obtained using the phylogeny from our data are presented.

### Analysis of selective pressures along trees: testing for an “age” and a “paralog” effect

**Figure 2:**
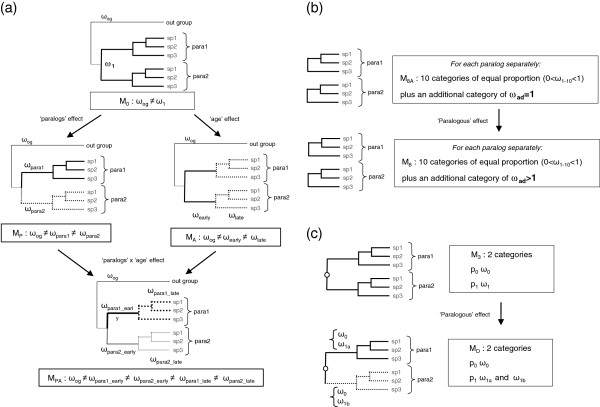
**Schematic representation of the codon models used.** (**a**) Models allowing dN/dS variation along lineages. Arrows indicate the questions addressed by the comparison between models (in (**a**), the arrows correspond to hierachical relationships). (**b**) Models allowing dN/dS variation along the gene. In M_8A_, ω follows a β distribution discretized into 10 categories of similar frequency (0 < ω_1–10_ < 1) and an additional category of ω is fixed at 1 (ω_ad_ = 1, accounting for neutral sites); M_8_ differs from M_8A_ only by the additional category of ω which is constraint to be superior to 1 (ω_ad_ > 1), to account for sites under positive selection. (**c**) Phylogenetic trees harbours two clades, one for each paralog (the outgroup is not represented here). Models are either specifying identical categories of dN/dS in both clades (M_3_), or allowing one category to take a different value in each clade (M_D_).

Comparing models with different constraints on the value of ω among branches of the tree allows testing evolutionary hypotheses (Figure 2a). The comparisons of M_A_ versus M_0_ and M_PA_ versus M_P_ test the “age effect” by contrasting early branches (when the duplication was young) and later branches. Similarly, the comparisons of M_P_ versus M_0_ and M_PA_ versus M_A_ test a “paralog effect” by examining the divergence between the two copies. Results of these tests are presented in Table 1 along with maximum-likelihood estimates of ω parameters of each model. When the *Pg11*/*Pg3* paralogs pair was analysed, both the sequences of *Pg11a* and *Pg11c* were considered for *Pg11*. For example, for testing the M_P_ model, both branches leading to *Pg11a* and *Pg11c* were considered for *Pg11*. For testing the M_A_ model, the branch between the node corresponding to the duplication between *Pg11* and *Pg3* and the node corresponding to the duplication between *Pg11a* and *Pg11c* (*i.e.* the ancestral *Pg11* gene) was considered as the late branch. Thus, any effects related to the duplication between *Pg11a* and *Pg11c* is considered only in the *Pg11a*/*Pg11c* paralogs pair analysis.

**Table 1:** Branch models: estimated parameters and log-likelihood ratio tests

**Paralog pair**	**Model**	**log*L***	**np**	**Branchs**	**ω**	**LRT**		***p*-value**
	M_0_	−2847.12	58	OG	0.05			
				*Lax*	0.08			
				OG	0.05			
	M_P_	−2846.50	59	*Lax2*	0.07	vs. M_0_	1.24	0.26
				*Lax4*	0.10			
				OG	0.05			
*Lax2*/*Lax4*	M_A_	−2840.62	59	*Lax*_early_	0.14	vs. M_0_	13.0**	0.00031
				*Lax*_late_	0.05			
				OG	0.05			
				*Lax2*_early_	0.19	vs. M_P_	13.8**	0.001
	M_PA_	−2839.60	61	*Lax4*_early_	0.11			
				*Lax2*_late_	0.04	vs. M_A_	2.04	0.36
				*Lax4*_late_	0.06			
	M_0_	−4127.20	62	OG	0.06			
				*Pg*	0.33			
				OG	0.06			
	M_P_	−4125.06	63	*Pg3*	0.25	vs. M_0_	4.28*	0.04
				*Pg11*	0.41			
				OG	0.06			
*Pg3*/*Pg11*	M_A_	−4124.78	63	*Pg*_early_	0.22	vs. M_0_	4.84*	0.03
				*Pg*_late_	0.38			
				OG	0.06			
				*Pg3*_early_	0.24	vs. M_P_	3.68	0.16
	M_PA_	−4123.22	65	*Pg11*_early_	0.21			
				*Pg3*_late_	0.29	vs. M_A_	3.12	0.21
				*Pg11*_late_	0.44			
	M_0_	−3604.36	40	OG	0.27			
				*Pg11*	0.44			
				OG	0.27			
	M_P_	−3603.75	41	*Pg11a*	0.38	vs. M_0_	1.22	0.27
				*Pg11c*	0.50			
				OG	0.27			
*Pg11a*/*Pg11c*	M_A_	−3604.19	41	*Pg11*_early_	0.50	vs. M_0_	0.34	0.57
				*Pg11*_late_	0.42			
				OG	0.27			
				*Pg11a*_early_	0.50	vs. M_P_	0.72	0.70
	M_PA_	−3603.39	43	*Pg11c*_early_	0.54			
				*Pg11a*_late_	0.35	vs. M_A_	1.60	0.45
				*Pg11c*_late_	0.49			

^*^Note. Models: for M_0_, ω is allowed to take a different value only in the branch of the outgroup (OG); for M_P_, M_A_ and M_PA_ ω is allowed to take a different values according to the tested effect, *i.e.* “paralogs”, “age” or combined as explained in Figure 1. np: number of free parameters; log*L*: log-likelihood; LRT: likelihood ratio test statistic between indicated models; one (respectively two) asterisk indicates that the probability of observing such an LRT or higher under the compared model is <0.05 (respectively <0.01), assuming that the LRT follows a χ^2^ distribution with the difference of free parameters between the compared models as the number of degrees of freedom.

Interestingly, the three paralogs pairs exhibited contrasted results. The *Lax2*/*Lax4* paralogs shows evidence for an age effect as shown by both a better fit of model M_A_ relative to M_0_ (LRT = 13.0, *p* = 0.00031) and M_PA_ versus M_P_ (LRT = 13.8, *p* = 0.001) tests. We observed a marked increase of ω in early branches (ω = 0.14 compared with ω = 0.05 for late branches in M_A_). No significant paralog effect was detected and both paralogs *Lax2* and *Lax4* seem to be evolving under purifying selection (ω = 0.08 in M_0_, ω = 0.07 and ω = 0.10 for *Lax2* and *Lax4* respectively in M_P_ model).

For the second paralog pair, *Pg11*/*Pg3*, both age (M_A_ vs. M_0_, LRT = 4.84, p = 0.03) and paralog effects (M_P_ vs. M_0_, LRT = 4.28, p = 0.04) are detected, but effects were weaker and marginally significant. The full model (M_PA_) did not provide a better fit relative to either the age or the copy models. The M_A_ model showed an increase of ω in late branches (ω = 0.38 as compared to 0.22 in early branches) and an increase of ω in *Pg11* (ω = 0.41 as compared to 0.25 in *Pg3*).

For the third and most recent pair of paralogous genes, *Pg11a*/*Pg11c*, no test was significant, suggesting that neither age nor paralog effects is playing a role or that the extent of nucleotide differences are too small for codon based models to have any power to detect heterogeneity in ω. The analysis shows that overall ω is markedly higher than in the other considered paralog pairs (ω = 0.44 for M_0_).

### Analysis of selective pressures along genes: testing for positive selection

In order to investigate how ω varies along genes and in particular if positive selection signatures occurred we used models in which ω is allowed to vary among sites on each gene (Figure 2b). As in analysis of selective pressures along trees, when *Pg11* gene is analysed, both sequences of *Pg11a* and *Pg11c* were considered. Since positive selection likely targets only a few amino acid positions, branch models used previously typically lack statistical power to detect positive selection as, in the branch model, ω is averaged over all the amino acid sites of the gene.

We compared the fit of models M_8A_ and M_8_. The likelihood ratio test of M_8_ against M_8A_ is a conservative test for positive selection, since the M_8A_ model can account for an excess of neutral sites. No sites under positive selection were found for *Lax2*, *Lax4* and *Pg3*. However the statistical test of M_8_ against M_8A_ was significant for *Pg11*, *Pg11a* and *Pg11c* (*p* = 1.4 10^-6^, 9.99 10^-3^ and 1.26 10^-4^ respectively) (Table 2), showing that positive selection targeted both copies of *Pg11*. The fitted ω values suggest that positive selection was stronger for *Pg11a* (ω = 11.61 at positively selected sites) than for *Pg11c* (ω = 4.45) but affected fewer sites (frequency of 0.02, equivalent to 1 site, for *Pg11a* versus 0.10, equivalent to 5 sites, for *Pg11c*). The amino acid site detected under positive selection in *Pg11a*, with a probability of 0.98, is at position 141 and corresponds to a Glycine (G) in the precursor of the protein in *M. truncatula* [GenBank:AES65910]. At this position, *M. ciliaris*, *M. polymorpha* and *M. ruthenica* have a Lysine (K), *M. rigiduloides* a Serine (S) and *M. coerulea* an asparagine (N). Five amino acid positions under positive selection were detected in *Pg11c*. None of these 5 amino acid positions is the same than that detected in *Pg11a*. Three amino acid positions had an estimated posterior probability to be under positive selection greater than 0.95: position 110 (a Glutamic acid, E), position 132 corresponding to a Glutamine (Q) and position 303 corresponding to a Threonine (T) (position on the precursor protein in *M. truncatula*, [GenBank:AES65907]). At position 110, *M. littoralis*, *M. tricycla* and *M. tornata* have an Aspartic acid (D), and *M. polymorpha* an Asparagine (N). At position 132, the Glutamine of *M. truncatula* changes for a Threonine (T) in *M. rigiduloides* and *M. noeana* and for an Alanine (A) in *M. polymorpha* and *M. coerulea*. Finally, at position 303, all the species have a Methionine (M), except *M. truncatula* which has a Threonine (T), *M. tricycla* and *M. tornata* a Leucine (L) and *M. polymorpha* a Lysine (K). The two other sites had a posterior probability of 0.997 and 0.996, on a Tryptophan (T) in position 161 and an Alanine (A) in position 270, respectively. At position 161, *M. rigiduloides*, *M. noeana*, *M. coerulea* and *M. ruthenica* have a Histidine (H), *M. polymorpha* a (R), and *M. rigidula* an Asparagine (N). Finally, at position 270, *M. littoralis*, *M. tricycla*, *M. rigiduloides* and *M. rigidula* have a Serine (S), and *M. noeana* a Glycine (G).

**Table 2:** Site models results: estimated parameters and log-likelihood ratio tests

**Gene**	**Model**	**np**	**log*L***	**Parameters**	**LRT**		***p*-value**
*Lax2*	M_8A_	35	−1600.22	p = 0.01 q = 2.86			
				ω_ad_ = 1 p_ad_ = 0.03			
	M_8_	36	−1600.19	p = 0.01 q = 3.00	vs. M_8A_	0.04	0.84
				ω_ad_ = 1.10 p_ad_ = 0.03			
*Lax4*	M_8A_	23	−1391.38	p = 6.30 q = 99.00			
				ω_ad_ = 1 p_ad_ = 0.00			
	M_8_	24	−1391.38	p = 6.30 q = 99.00	vs. M_8A_	0.00	1
				ω_ad_ = 1.00 p_ad_ = 0.00			
*Pg3*	M_8A_	23	−1616.24	p = 4.89 q = 99.0			
				ω_ad_ = 1.00 p_ad_ = 0.24			
	M_8_	24	−1615.18	p = 0.13 q = 0.40	vs. M_8A_	2.11	0.15
				ω_ad_ = 4.88 p_ad_ = 0.01			
*Pg11*	M_8A_	39	−2221.99	p = 2.16 q = 99.00			
				ω_ad_ = 1 p_ad_ = 0.36			
	M_8_	40	−2210.38	p = 0.01 q = 20.01;	vs. M_8A_	23.22**	1.44 10^-6^
				ω_ad_ = 6.30 p_ad_ = 0.04			
*Pg11a*	M_8A_	19	−1388.97	p = 2.32 q = 89.36			
				ω_ad_ = 1.00 p_ad_ = 0.35			
	M_8_	20	−1385.65	p = 0.47 q = 0.96	vs. M_8A_	6.64**	9.99 10^-3^
				ω_ad_ = 11.61 p_ad_ = 0.02			
*Pg11c*	M_8A_	21	−1519.41	p = 0.01 q = 2.54			
				ω_ad_ = 1.00 p_ad_ = 0.32			
	M_8_	22	−1512.06	p = 0.01 q = 0.05	vs. M_8A_	14.69**	1.26 10^-4^
				ω_ad_ = 4.45 p_ad_ = 0.10			

Note. Models are M_8A_, ω following a β distribution discretized into 10 categories of similar frequency (0 < ω_1–10_ < 1) plus an additional category of ω_ad_ = 1, accounting for neutral sites; M_8_ differs from M_8A_ only by the additional category of ω which is constraint to be superior to 1 (ω_ad_ > 1), to account for sites under positive selection.

Parameters are frequencies and values of ω for M_8A_ and M_8_, p and q are the parameters in β distribution; for M_8A_ and M_8_ p_ad_ and ω_ad_ are the frequencies and values of additional class of ω; NB ω_ad_ is fixed equal to one in M_8A._ np: number of free parameters; log*L*: log-likelihood; LRT: likelihood ratio test statistic between indicated models; one (respectively two) asterisk indicates that the probability of observing such an LRT or higher under the compared model is <0.05 (respectively <0.01), assuming that the LRT follows a χ^2^ distribution with the difference of free parameters between the compared models as the number of degrees of freedom.

### Selective pressures along branches and sites of each paralog: testing for a “paralog” effect

In the third model we used, the clade model M_D_[33], ω varies among sites (with either two or three categories) and selective pressure at one class of sites is allowed to differ in the two clades of the phylogeny (Figure 2c). We tested the significance of M_D_ models, with two (or three) categories of sites, compared to null M_3_ models (discrete model), which assume that two (or three) classes of sites are evolving under different levels of selective pressures, but without difference between clades. As in the previous sections, when the *Pg11*/*Pg3* paralogs pair was analysed, both the sequences of *Pg11a* and *Pg11c* were considered for *Pg11*.

For the three paralogous gene pairs studied, models M_D_ for which one class of ω is allowed to differ between paralogous gene clades were significantly better than null models M_3_ in which no variation between clades is allowed (Table 3). For the *Lax2* and *Lax4* paralogs tests comparing M_D_ and M_3_ were significant when either two or three categories of ω were considered. For the other two pairs of paralogs, the test comparing M_D_ and M_3_ was significant only when both models were defined with two categories of ω. These results revealed, for each pair, the presence of sites evolving under divergent selective pressures between the paralogous gene clades.

**Table 3:** Branch-site models: estimated parameters and log-likelihood ratio tests

	**Model (k)**	**np**	**Log*L***	**LRT**		***P*-value**	**prop**	**clade**	**ω**
	M_3_ (2)	58	−2375.94						
*Lax2*/*Lax4*	M_D_ (2)	59	−2369.54	vs. M_3_ (2)	12.8**	3.47 10^-4^	0.76		0.00
							0.24	*Lax2*	0.15
								*Lax4*	0.52
	M_3_ (3)	60	−2375.94						
	M_D_ (3)	61	−2361.85	vs. M_3_ (3)	28.17**	1.11 10^-7^	0.77		0.005
							0.03		0.97
							0.20	*Lax2*	0.55
								*Lax4*	0.97
	M_3_ (2)	61	−3411.03						
*Pg11*/*Pg3*	M_D_ (2)	62	−3407.95	vs. M_3_ (2)	6.16*	0.01	0.68		0.09
							0.32	*Pg3*	0.74
								*Pg11*	1.35
	M_3_ (3)	63	−3400.58						
	M_D_ (3)	64	−3399.59	vs. M_3_ (3)	1.98	1.16			
	M_3_ (2)	40	−2216.23						
*Pg11a*/*Pg11c*	M_D_ (2)	41	−2214.01	vs. M_3_ (2)	4.45*	0.03	0.79		0.12
							0.21	*Pg11a*	1.54
								*Pg11c*	3.13
	M_3_ (3)	42	−2210.38						
	M_D_ (3)	43	−2209.37	vs. M_3_ (3)	2.01	0.16			

Note. Models: for M_3_ (discrete model), ω is free to take the number of values indicated in brackets (k); these values are homogenous in all branches of the tree; for M_D_, as explained in Figure 1 (b), one category of ω is allowed to differ between the two paralogous genes clades of the tree [33]. np: number of free parameters; log*L*: log-likelihood; LRT: likelihood ratio test statistic between indicated models; one (respectively two) asterisk indicates that the probability of observing such an LRT or higher under the compared model is <0.05 (respectively <0.01), assuming that the LRT follows a χ^2^ distribution with the difference of free parameters between the compared models as the number of degrees of freedom.

For *Lax2*/*Lax4*, none of the ω values was larger than 1, consistently with the result of the M_8_ versus M_8A_ comparison. The model with three categories indicates that more than 77% of amino acid positions are very strongly constrained (ω very close to 0). The other two categories are allowed to vary between the two clades. For *Lax2* a small proportion of sites is neutrally evolving (ω ~ 1) and the rest is mildly constrained (ω = 0.55), whereas in *Lax4* both categories are effectively neutral (ω = 0.97).

For *Pg11*/*Pg3* and *Pg11a*/*Pg11c*, the category of sites fixed across clades were also found to be under purifying selective pressure (ω = 0.09 and 0.12, respectively). When 2 categories of ω were considered, M_D_ was significantly better than the null model M_3_ (LRT = 6.16, *p* = 0.01 and LRT = 4.45, *p* = 0.03 for the *Pg11*/*Pg3* and *Pg11a*/*Pg11c* paralogs pairs respectively). The category of sites allowed to differ in M_D_ model had a proportion of 32% and appeared to be nearly neutrally evolving in *Pg3* (ω = 0.74) but under positive selection in *Pg11* (ω = 1.35), as found with the M_8_ model. Concerning the *Pg11a*/*Pg11c* paralogous gene pair and as previously detected with site models, the M_D_ model revealed that positive selection occurs for the *Pg11a* gene and for the *Pg11c* gene (ω = 1.54 and 3.13 respectively), but in addition M_D_ actually detected a difference in the rate of positive selection between the paralogous copies, which appeared to be stronger in *Pg11c*.

## Discussion

In this paper we examined patterns of molecular evolution of three paralogous gene pairs, in order to detect signatures of post-duplication functional divergence. We chose a time scale that allows analysing patterns of natural selection by examining patterns of nucleotide substitution of protein-coding sequences. With that aim, we focused on three sets of paralogs from the *Medicago truncatula* genome, *Lax2*/*Lax4*, *Pg3*/*Pg11* and *Pg11a*/*Pg11c*. The duplications leading to these sets of paralogs occurred before the radiation of the 17 species studied but are still recent, as the three set of paralogs, *Lax2*/*Lax4*, *Pg3*/*Pg11* and *Pg11a*/*Pg11c,* exhibit still 83, 72 and 88% nucleotide identity, respectively. Furthermore, we selected genes that are putatively involved in symbiotic functions, considering that interspecific interactions can involve both evolution of novelty (especially in the case of the legume-rhizobium symbiosis which evolved relatively recently) and co-evolutionary phenomena that are detectable through signatures of positive selection.

Models describing the evolutionary fate of duplicated genes once the duplication is fixed in the species suppose different forms of selective pressures [11]. First, according to the neofunctionalization model, *i.e.* evolution of a new function through functional divergence of one of the duplicated copies, selective pressures are expected to be asymmetrical between paralogs [1]. The copy fulfilling the ancestral function is expected to remain under purifying selection while the other copy is expected to experience a short period of relaxed constraint and then positive selection driving the acquisition of its new function. Second, the subfunctionalization model envisions the fixation of complementary degenerative mutations [12]. Under this model, relaxation of purifying selection is expected during the period of functional redundancy, and may allow the fixation of at least two complementary degenerative mutations (one in each gene). When both copies are jointly required to fulfil the ancestral gene function, purifying selection is still expected to be prevalent to maintain both copies. Although both models have been functionally validated, they are not exclusive and more complex scenarios combining the steps cited previously have been devised [15,25].

For all three studied paralogous gene pairs, the two copies exhibit different regimes of selection. This result suggests that these paralogous gene pairs have undergone at least some functional differentiation. Three different tests were used to qualify selective pressures governing the paralogs. The first one contrasted the average ω between paralog clades of the phylogeny and yielded significant differences only for *Pg11*/*Pg3* (Table 1). The second test is specifically designed to detect positive selection affecting only a few sites of the sequence. We found signatures of positive selection in both *Pg11a* and *Pg11c* copies, and in *Pg11* (Table 2). Finally, the clade model (Table 3) is a combination of branch and site models and allows investigating specifically the presence of sites evolving under divergent selective pressures between the paralogous genes and quantify its proportion. The clade model (M_D_) detected a significant increase of ω in *Pg11* due to the occurrence of positive selection, as detected by the site model M_8_. For the paralogous pair *Pg11a/Pg11c*, branch models failed to detect any difference in selective pressure. Model D is more detailed and allows showing that sites under positive selection actually experience a stronger positive pressure in *Pg11c* than in *Pg11a*. *Lax2* is the subject of an intense purifying selection whereas *Lax4* harbours some sites (20% of sites) evolving quasi neutrally (ω = 0.97). The combination of these different tests provides a more complete picture of the selective pressures at work on each set of paralog. Since each single test addresses a single hypothesis, the comparison of several complementary tests allows acquiring a more complete picture. However, the clade model, which accounts for both variation of ω among branches and amino acid position, appears as the most informative for qualifying changes of selective constraint during duplicated genes evolution [33]. The only drawback is that it does not test formally for positive selection.

We observed that the *Pg3* and *Pg11c* gene copies were pseudogenes in several species: in *M. littoralis* and *M. tricycla* for *Pg3* and in *M. tornata*, *M. rigidula* and *M. polymorpha* for *Pg11c*. Since the three genes are present and potentially functional in, at least, four other species among those studied, we can hypothesise that the mutations affecting the function of these gene copies occurred, in some phylum, after the two successive rounds of duplications leading to the presence of three copies. This observation suggests that redundancy between copies is sufficient to have allowed the loss of one copy in several species.

Functional redundancy generated by multiple copies also implies periods of relaxed selection pressures, except if duplication itself is advantageous as it is the case, for instance, for a positive dose effect of copy number [11]. Redundancy is expected to occur with a larger probability when divergence between copies is slowed as it is the case of gene conversion [34]. The phylogenetic miss positioning we observed for four genes copies (Figure 1) may be explained by gene conversion. One way to test this hypothesis would be to sequence other individuals of *M. tornata* for example, in order to see if we could detect shared polymorphism between copies, which is a signature of gene conversion [11].

We detected sites under positive selection in *Pg11* but not in *Pg3*. Rodriguez-Llorente *et al.*[26] suggested that *Pg3* has been recruited by symbiosis after a duplication affecting an ancestral pollen-specific gene. The authors suggested that the modifications occurred essentially in the promoter region. Our results show that positive selection targeted both copies of *Pg11* independently, possibly indicating the evolution of novel gene function. The polygalacturonase family contains members in organisms as distantly related as plants and eubacteria. In plants this gene family has been expanding dramatically through rounds of whole-genome duplications, segmental duplications and tandem duplications (66 and 59 copies in *Arabidopsis thaliana* and rice respectively) [35]. The high level of expansion of this family, generating periods of high redundancy, was probably accompanied by pseudogenization events, equivalent to those we detected in the *Medicago* genus. However as expression patterns are diverse between members of the family [35] subfunctionalization events were probably involved in the overall high retention rate of functional genes, notable in this family. Functional divergence among members of large gene families may also be driven by positive selection. Main examples in plants are disease resistance genes [36], transcription factors [37] or genes involved in development [38]. In our study, positive selection is detected in *Pg11*, resulting from the cumulative effects of positive selection in both *Pg11a* and *Pg11c*, the more recent duplicated gene pair we studied. Actually, this mode of selection does correspond to neither neofunctionalization nor subfunctionalization in their stricter definition. Subfunctionalization does not predict positive selection in either copy, while neofunctionalization predicts positive selection in only one copy (if detectable). Both copies could be under positive selection because they inherited, from the ancestral *Pg11* gene, functions that imply regime of positive selection. Alternatively, neo-functionalization could involve adaptive differentiation of both copies (to avoid functional overlap), that would mediate adaptive evolution of both copies. Selection targets different sites in *Pg11a* and *Pg11c* and the strength of positive selection is different between them (Table 3). This observation is compatible with both models.

According to the clade models, the paralogs *Lax2* and *Lax4* experience different modes of selection. Both genes are mainly under purifying selection. Interestingly no pseudogenes were detected in *Lax2* or in *Lax4*. The redundancy stage subsequent to the duplication generating *Lax2* and *Lax4* is not detectable anymore and may have been shorter than in *Pg* gene family. However, *Lax4* appeared to be slightly, but significantly, less constrained than *Lax2*. According to the clade models (with 2 or 3 classes of sites, Table 3) a relaxation of constraint is observed for about 20% of the sites for *Lax4* relative to *Lax2*. This means either that *Lax4* acquired a function that implies less functional constraints or that both genes underwent subfunctionalization in such a way that the protein sequence of *Lax4* is less constrained. Currently, the precise functions of *Lax2* and *Lax4* are not known. Both paralogs are expressed in shoot and roots of nodulating plants of *M. truncatula*. *Lax2* is found in Expressed Sequence Tag (EST) libraries built from different tissues (2 in early seed development, 2 in flowers, early seeds, late seeds and stems, 2 in mixed root and nodules, 1 in nematode-infected roots, in developing flowers and phosphate-starved leaf). *Lax4* is not found in EST libraries but expression of *Lax4* was detected in shoots and roots of nodulating plants of *M. truncatula*[39].

The models contrasting ω in different branches allowed testing transient relaxation of purifying selection predicted to occur immediately after duplication. A significant increase of ω was detected in basal branches of the *Lax2*/*Lax4* phylogeny. The opposite trend was detected for the *Pg11*/*Pg3* pair, where purifying selection appeared to be actually weaker in late branches than in early branches, particularly for *Pg11* (ω = 0.44). However, the value of ω in late branches was likely biased by the occurrence of positive selection in *Pg11*, because branch models average over all sites.

## Conclusions

This study illustrates the multiplicity of mechanisms governing the evolutionary fate of duplicated genes and, in particular, the relative age of the duplication. Analysis of nucleotide substitution rates in gene coding sequence can discriminate between qualitative phenomenon (occurrence of positive selection) or quantitative differences (levels of ω between clades and its variation among branch and sites). Further studies of the factors governing evolution of duplicated genes will benefit from taking into account features of the evolution of gene families involving successive rounds of duplications.

## Methods

### Plant material

One accession was selected in sixteen diploid species of the *Medicago* genus: *M. arabica, M. ciliaris, M. carstiensis, M. coerulea, M. laciniata, M. littoralis, M. marina, M. noëana, M. orbicularis, M. polymorpha, M. rigidula, M. rigiduloides, M. ruthenica, M. sauvagei, M. tornata, M. tricycla*. Accession numbers, geographic location and mating systems are presented in Additional file 2.

### Selection of duplicated genes

Genes were chosen on the basis of the *Medicago truncatula* line A17 whole genome sequence [28]. We selected two multigenic families meeting exhibiting recent rounds of duplications and involved in symbiosis-related functions. First, polygalacturonases (PG) form a gene family that is ubiquitous in the plant kingdom. These proteins are involved in the degradation of polysaccharides found in higher plants cell walls. The gene *Pg11* is involved in pollen tube elongation in *M. truncatula* and is located on chromosome 2. *Pg3*, located on chromosome 5 in *M. truncatula*, has been shown to be involved in the tip growth of the infection thread during the establishment of the symbiosis with nitrogen-fixing bacteria *Sinorhizobium* sp. [26]. We also identified a more recent tandem duplication of *Pg11*, resulting in the paralogs *Pg11a* and *Pg11c*. The pairs *Pg3Pg11c* and *Pg11aPg11c* exhibit respectively 72 and 88% nucleotide sequence identity, and 62 and 81% amino acid sequence identity.

Second, we chose family of auxin efflux carrier, *Lax* (Like-*Aux1*), for which five members have been identified in *Medicago truncatula*[39]. Auxin is generally involved in the control of tissue growth and in particular during the development of nodules, the symbiotic organ hosting *Sinorhizobium* symbionts [40]. Auxin is synthesized in aerial organs (leaves and shoot apex) and is directionally transported. As a result, auxin carriers such as LAX proteins play an important role in auxin-controlled processes. We chose to study the youngest paralogous gene pair *Lax2Lax4* that presents 83% of nucleotide identity, and 87% of amino acid identity. The sequence accession numbers are AY115843 and AY115844 respectively for *Lax2* and *Lax4*.

### Sequencing

To amplify specifically the coding region of each paralogous gene, we defined specific and non-specific primers. Non-specific primers were defined using the common sequence of both paralogous gene for each pair (*i.e.* not allowing to amplify separately each paralogs), whereas copy-specific primers were defined using polymorphism between the paralogs, available in the reference *Medicago truncatula* genotype A17. In a first step, specific primers combinations were used to amplify specifically each paralogs. Then, sequencing was performed using specific and/or non-specific primers. The primer sequences and their position on the genomic sequences of the five genes are available in Additional file 3 and Additional file 4 respectively. As divergence between species was often the cause of unsuccessful amplifications, several copy-specific primer pairs were defined to increase the chances of amplification in the sixteen studied species. Additional amplification rounds were performed to close sequencing gaps. For the most recent paralogs pair (*Pg11a*-*Pg11c*), the sequences obtained were labelled according to the primer combinations used for amplification and sequencing: when using primers designed for *Pg11a* (respectively *Pg11c*) , the sequencing product was qualified as ‘*Pg11a*’ copy (respectively ‘*Pg11c*’ copy).

Most sequences were obtained from genomic DNA, except for *Lax2*, which was sequenced from cDNA due to its large size. DNA extraction and genomic DNA amplifications and sequencing were performed as described in [41]. Total RNA was extracted from fresh leaves with a TRI REAGENT (T9424, Sigma®) buffer. Reverse transcription was done using the Reverse Transcription System kit from Promega®. Amplification and sequencing from cDNA were then performed as for genomic DNA. Chromatograph assembly and alignment were performed using programs of the Staden package v1.5 [42]. Visual inspection and correction of base calling and alignment were performed at this stage. The sequence editor Artemis v9 [43] was used to validate the reading frame and detect eventual frame shifts and/or premature stop codon mutation.

### Outgroups

The *Medicago truncatula* sequence of gene *Lax1* [GenBank:AY115841] was used as outgroup to root the *Lax2Lax4* pair phylogeny. *Lax1* diverged from *Lax2* and *Lax4* through a more ancient duplication [39]. Following the phylogenetic tree of the plants PG and endoglucanases published by Rodriguez-Llorente [26], we selected a PG coding sequence from *Salix gilgiana* [GenBank:AB029458] as outgroup for the Pg3-*Pg11a*/*c* phylogenetic tree. The *M. truncatula* copy of *Pg3* was used as outgroup for the *Pg11a- Pg11c* pair phylogeny.

### Phylogenetic analysis

Maximum-likelihood phylogenetic trees were inferred using the PHYML program [44]. Maximum-likelihood analyses were conducted under the GTR molecular substitution model. Site to site variation in substitution rate was modeled by estimating the proportion of invariant sites and assuming that rates among the remaining sites were gamma distributed (4 categories were used to discretize the gamma distribution). The confidence level of each node was estimated using 100 bootstrap repetitions. Nucleotide and amino acid alignments of *Lax* genes are available in Additional file 5 and Additional file 6 respectively. Nucleotide and amino acid alignments of *Pg* genes are available in Additional file 7 and Additional file 8 respectively.

Variation in substitution rates was analyzed using codon substitution models where the parameter ω is defined as the ratio of non-synonymous (dN) to synonymous (dS) substitution rates [45]. We used eight models that make different assumption regarding variation of ω (Figure 2) in the phylogeny of each pair of paralogs. The first four models account of variation of ω among branches of the phylogeny [46] (Figure 2a). Model M_0_ assumes a single ω value for both paralogs. Model M_P_ allows a “paralog effect” by assigning a different ω for each paralog clade in the tree. Model M_A_ allows an “age effect” and assigns a single ω for the basal (ancestral) branch of both paralogs clades and a different ω to all other (more recent) branches within both clades. Model M_PA_ allows for both levels of variation. All four models above are also specifying a specific ω parameter value on the branch leading to the outgroup of each paralog phylogeny. Total numbers of ω parameters are 2 for M_0_, 3 for M_A_ and M_P_, and 5 for M_PA_.

Next, two models allowing for variation of ω among sites, but not among branches of the phylogenetic tree, and that are designed specifically to detect positive selection were used (Figure 2b) [47]. M_8A_ assumes that a fraction of the sites experience purifying selection of varying intensity by assuming that ω omega values follow a beta distribution (0 < ω < 1). The remaining fraction of the site are assumed to evolve neutrally (ω = 1). M_8A_ was used as null model for detecting positive selection by comparing its fit with M_8_ in which the additional category of ω is free to take any value above 1 (positive selection). The comparison of M_8_ versus M_8A_ provides a (likelihood ratio) test for the occurrence of positive selection (identified when at least some sites exhibit a ω > 1). These two models were fitted separately to each paralog in the tree and excluding the outgroup, in order to detect positive selection occurring specifically on each copy.

The last two models are so called branch-site models that are combining variation of omega both among amino acid positions of the alignment and between different clades of the phylogeny, in our case each paralog clade (Figure 2c). These models allow testing variation of selective constraints between paralogous copies. Outgroup sequences are not considered in this analysis. The null model M_3_ allows either two or three rate categories that are homogeneous along the tree. Model M_D_ (model D in [33]) allows selective pressure at one class of sites to differ in different clades of the phylogeny. Applied to our case, it is allowed to differ in each clade of paralogous gene (Figure 2c).

Maximum likelihood estimation of all model parameters was performed using the codeml software of the PAML package [48]. The different pairs of models are nested and were compared using likelihood ratio tests (LRTs).

## Abbreviations

Pg: Polygalacturonase; Lax: Like*-Aux1* (auxin efflux carrier); LRTs: Likelihood ratio tests; OG: Outgroup; EST: Expressed sequence tag.

## Competing interests

The authors do not have any kind of financial or non-financial competing interest to declare in relation to this manuscript.

## Authors’ contributions

NC and JR conceived and designed research, JHH, IH and AW acquired data, JHH and NC processed data, JHH, NC and SDM analyzed data and NC, JHH, JR, SDM and TB wrote the paper. All authors read and approved the final manuscript.

## Supplementary Material

Additional file 1 **Sequencing results.** Table in PDF format presenting sequencing results for the five genes on the 17 species and GenBank accession numbers. Lengths are indicated in base pairs. The percentage that each sequence represents relative to the complete alignment is indicated in brackets when less than 100%. “*na*” and “*ns*” are indicated when an amplification failed and when the sequence was too short to be included in the analyses, respectively. Four sequences presented either point mutations resulting in a stop codon (*Pg11c* of *M. laciniata*), or a deletions inducing a frame shift in the coding sequence (*Pg11c* of *M. ciliaris*) or resulting in the appearance of a premature stop codon (for three sequences: *Pg11c* of *M. orbicularis* and *Pg3* of *M. littoralis* and *M. tricycla*) are indicated by “*pseudo*”. Sequences with an unexpected position in the phylogeny are noted as “*phylo_excluded*”. Click here for file

Additional file 2 **List of species used.** Table in PDF format with list of sample used, germplasm accession number, life history, geographical area and ploidy level. Click here for file

Additional file 3 **List of primers used.** Table in PDF format with names and sequences of primers used for amplification and sequencing. Click here for file

Additional file 4 **Schematic representation of genes and primers positions.** Figure in PDF format with schematic representation of the intron/exon structure of the 5 sequenced genes on *M. truncatula* (A17) and position of the primers used for the amplification and sequencing, names and sequences of primers used for amplification and sequencing. Click here for file

Additional file 5 **Lax gene Nucleotide alignment.** Nucleotide alignment of *Lax* genes in phyml format. Click here for file

Additional file 6 ***Lax*gene amino acid alignment.** Amino acid alignment of *Lax* genes in phyml format. Click here for file

Additional file 7 ***Pg*gene nucleotide alignment.** Nucleotide alignment of *Pg* genes in phyml format. Click here for file

Additional file 8 **Pg gene amino acid alignment.** Amino acid alignment of *Pg* genes in phyml format. Click here for file
