# Supplementary material for: Contrasted patterns of selective pressure in three recent paralogous gene pairs in the Medicago genus (L.)
Source: BMC Evol Biol. 2012 Oct 1;12:195. doi: 10.1186/1471-2148-12-195 (PMC3517903; doi:10.1186/1471-2148-12-195)
Supplement: Additional file 1 — Sequencing results. Table in PDF format presenting sequencing results for the five genes on the 17 species and GenBank accession numbers. Lengths are indicated in base pairs. The percentage that each sequence represents relative to the complete alignment is indicated in brackets when less than 100%. “na” and “ns” are indicated when an amplification failed and when the sequence was too short to be included in the analyses, respectively. Four sequences presented either point mutations resulting in a stop codon (Pg11c of M. laciniata), or a deletions inducing a frame shift in the coding sequence (Pg11c of M. ciliaris) or resulting in the appearance of a premature stop codon (for three sequences: Pg11c of M. orbicularis and Pg3 of M. littoralis and M. tricycla) are indicated by “pseudo”. Sequences with an unexpected position in the phylogeny are noted as “phylo_excluded”. [file 1471-2148-12-195-S1.doc]

### Additional file 4 – Sequencing results

Table presenting sequencing results for the five genes on the 17 species and GenBank accession numbers. Lengths are indicated in base pairs. The percentage that each sequence represents relative to the complete alignment is indicated in brackets when less than 100%. ‘*na*’ and ‘*ns*’ are indicated when an amplification failed and when the sequence was too short to be included in the analyses, respectively. Four sequences presented either point mutations resulting in a stop codon (*Pg11c* of *M. laciniata*), or a deletions inducing a frame shift in the coding sequence (*Pg11c* of *M. ciliaris*) or resulting in the appearance of a premature stop codon (for three sequences: *Pg11c* of *M. orbicularis* and *Pg3* of *M. littoralis* and *M. tricycla*) are indicated by ‘*pseudo*’. Sequences with an unexpected position in the phylogeny are noted as ‘*phylo_excluded*’.

| ***Species*** | ***Pg11a*** | ***Pg11c*** | ***Pg3*** | ***Lax2*** | ***Lax4*** |
| --- | --- | --- | --- | --- | --- |
| *littoralis* | 720  JN635642 | 720  JN635651 | *pseudo*  - | 798  JN635664 | 798  JN635679 |
| *tricycla* | 720  JN635643 | 720  JN635652 | *pseudo*  - | 798  JN635665 | 650 (77)  JN635680 |
| *truncatula* | 720  JN635644 | 720  AC187464* | 714  AJ620946 | 798  AY115843 | 798  AY115844 |
| *tornata* | *phylo_excluded*  JN635641 | 720  JN635653 | 708  HQ737838 | 798  HQ736585 | 614 (77)  HQ736701 |
| *ciliaris* | 426 (67)  JN635645 | *pseudo*  - | *na*  *-* | 798  JN635666 | *ns*  *-* |
| *rigiduloides* | 720  JN635646 | 720  JN635654 | 708  JN635632 | 798  JN635667 | 609 (76)  JN635681 |
| *rigidula* | 720  JN635647 | 723  JN635655 | 708  JN635633 | 798  JN635668 | 798  JN635685 |
| *laciniata* | *na*  *-* | *pseudo*  - | 708  JN635634 | 798  JN635669 | 759 (95)  JN635686 |
| *noëana* | *na*  *-* | 720  JN635656 | 708  JN635635 | 798  JN635670 | 798  JN635687 |
| *arabica* | *na*  *-* | *ns*  JN635657 | 708  JN635636 | 798  JN635671 | *ns*  *-* |
| *polymorpha* | 456 (70)  JN635648 | 720  JN635658 | *na*  *-* | 798  JN635672 | 798  JN635682 |
| *orbicularis* | *na*  *-* | *pseudo*  - | *na*  *-* | 798  JN635673 | 486 (61)  JN635683 |
| *sauvagei* | *na*  *-* | *phylo_excluded*  JN635659 | 708  JN635637 | 798  JN635674 | *ns*  *-* |
| *carstiensis* | *na*  *-* | *phylo_excluded*  JN635660 | 708  JN635638 | 798  JN635675 | *na*  *-* |
| *coerulea* | 720  JN635649 | 720  JN635661 | 708  JN635639 | 798  JN635676 | *ns*  *-* |
| *ruthenica* | 352 (59)  JN635650 | *phylo_excluded*  JN635662 | *ns*  *-* | 798  JN635678 | *na*  *-* |
| *marina* | *na*  *-* | 723  JN635663 | 708  JN635640 | 798  JN635677 | 798  JN635684 |
| *total nb of accessions* | 10 | 13 | 11 | 17 | 11 |
| *alignment length* | 729 | 729 | 729 | 798 | 798 |

* position 103679 to 105217
