# Supplementary material for: Contrasted patterns of selective pressure in three recent paralogous gene pairs in the Medicago genus (L.)
Source: BMC Evol Biol. 2012 Oct 1;12:195. doi: 10.1186/1471-2148-12-195 (PMC3517903; doi:10.1186/1471-2148-12-195)
Supplement: Additional file 2 — List of species used. Table in PDF format with list of sample used, germplasm accession number, life history, geographical area and ploidy level. [file 1471-2148-12-195-S2.doc]

### Additional file 1 – List of species used

Table with list of sample used, germplasm accession number, life history, geographical area and ploidy level.

| Species | Sample used | Germplasm accession | Life history | Geographical area | Ploidy (2n=) |
| --- | --- | --- | --- | --- | --- |
| *M. arabica* | F34022 | L00909 | annual selfer | Mediterranean basin | 16 |
| *M. carstiensis* |  | LCars | perennial selfer | East Adriatic | 16 |
| *M. ciliaris* | DZA204 | L00897 | annual selfer | Mediterranean basin | 16 |
| *M. coerulea* |  | LCoer | perennial outcrosser | East Asia | 16 |
| *M. laciniata* | DZA239 | L00904 | annual selfer | Mediterranean basin | 16 |
| *M. littoralis* |  | L00122 | annual selfer | Mediterranean basin | 16 |
| *M. marina* | CarnonR96 | LMari | perennial outcrosser | Mediterranean basin | 16 |
| *M. noeana* |  | L00908 | annual selfer | Iraq, Turkey | 16 |
| *M. orbicularis* |  | L00913 | annual selfer | Mediterranean basin | 16 |
| *M. polymorpha* | F34003 | L00911 | annual selfer | worldwide | 14 |
| *M. rigidula* | ES024 | L00901 | annual selfer | Mediterranean basin | 16 |
| *M. rigiduloides* | ICISIS | L00899 | annual selfer | Asia | 16 |
| *M. ruthenica* |  | LRuth | perennial outcrosser | East Asia | 16 |
| *M. sauvagei* |  | L00928 | annual selfer | Morocco | 16 |
| *M. tornata* |  | L00750 | annual selfer |  | 16 |
| *M. truncatula* | A17 | L00738 | annual selfer | Mediterranean basin | 16 |
| *M. truncatula ssp. tricycla* |  | L00220 | annual selfer | Mediterranean basin | 16 |
