# Supplementary material for: Contrasted patterns of selective pressure in three recent paralogous gene pairs in the Medicago genus (L.)
Source: BMC Evol Biol. 2012 Oct 1;12:195. doi: 10.1186/1471-2148-12-195 (PMC3517903; doi:10.1186/1471-2148-12-195)
Supplement: Additional file 3 — List of primers used. Table in PDF format with names and sequences of primers used for amplification and sequencing. [file 1471-2148-12-195-S3.doc]

### Additional file 2 – List of primers used

Table with names and sequences of primers used for amplification and sequencing.

| **Name** | **Sequence** | **notes** |
| --- | --- | --- |
| Lax2_1F | TTTGCATTTATTTGGATCTGGGC |  |
| Lax2_1R | TGTAACAGCATGTCCACCAAA |  |
| Lax2_2F | TGGTACCTTACTATAGCATCA | *Lax2* specific |
| Lax2_2R | AGTAAGCATATGAGCAGCAGA | *Lax2* specific |
| Lax2_3F | AGCATTTGTCTAAGGGCACTTG |  |
| Lax2_3R | ACTTGCACAACACACAGCCAGC |  |
| Lax2_4F | AGAGATTATGCACGCCATGTGG |  |
| Lax2_4R | AGTATAACAGCACCATCACGCC |  |
| Lax2_5R | AGAAAGGTGGCTTTTCAGCAGCA |  |
| Lax2-5F | TGATTTTGGAAATAATGTTGCAG | *Lax2* specific |
| Lax2-6F | GCCACAAAAACAAGGAGAGG | *Lax2* specific |
| Lax2-6R | TTCCAATATGGACCCAGTAAC | *Lax2* specific |
| Lax2-7R | ACACTGTAACAGCATGTCCACCA | *Lax2* specific |
| Lax2-8R | CTTTCGTGCGCTGGCTTTTCGG | *Lax2* specific |
| Lax2-9R | AACCAAACGGACCCTGAACGACT | *Lax2* specific |
| Lax2-10R | AACCAAACAAACCCTAAACGACG | *Lax2* specific |
| Lax2-7F | AGTTCATCACATTTGGTTTTGCG | *Lax2* specific |
| Lax2-8F | GTAATAGGAATGCATGACACAAG | *Lax2* specific |
| Lax2-11R | TCAATGATGAAGAGCATGAGGA | *Lax2* specific |
| Lax2-12R | ACACCAAATGAAGGGGTTGAGT | *Lax2* specific |
| Lax2-13R | TGAGTTTGTCAGAATCACGAAAC | *Lax2* specific |
| Lax2-14R | GTATAAGATTTCAATCAACATC | *Lax2* specific |
| Lax2-9F | TAGCATCAATCGTTCATGGCC | *Lax2* specific |
| Lax2-10F | ACGATTCCTTCTGCCACTGCT | *Lax2* specific |
| Lax2-11F | TCTCTTAGTAATGCCACATGCTC | *Lax2* specific |
| Lax2-12F | TGGAAGAGAAGAAGAGGTGGA | *Lax2* specific |
| Lax2-15R | ACAAGAAGAGCTCCAACAGCAG | *Lax2* specific |
| Lax2-16R | CATTTGAACCAAACGGACCCT | *Lax2* specific |
| Lax2-17R | TCCAAACAAAACCAACCTTAG | *Lax2* specific |
| Lax2-18R | ACGCCATCCATTTTTAGGTAGG | *Lax2* specific |
| Lax4_1F | GTTATATGGATCTGGGGTTGTGT |  |
| Lax4_1R | ACTGTCACAGCATGTCCACCG |  |
| Lax4_2F | TACACTCTGGTCCAACAAAGC |  |
| Lax4_2R | TCTATAAGTGAGCATATGAGCT |  |
| Lax4_3F | AGTATTTGTTTGAGGGCACTTGT |  |
| Lax4_3R | ACAAACTATGCAAAACCCAACCA |  |
| Lax4_4F | TGGTTTGAAGTTCTTGATGGA |  |
| Lax4_4R | CTTGCACATGCTATAAGCTGAAT |  |
| Lax4_5F | AGAGATTATGCATGCAATGTGG |  |
| Lax4_5R | TGAGCATGAGGATAACGGCAGC |  |
| Lax4_6F | TCATGGTATTAGAGGGGCCTC | *Lax4* specific |
| Lax4_6R | ACATTGCCTTGGTGACCCTACCC | *Lax4* specific |
| Lax4-7F | TGGTACACTCTGGTCCAACAAAG | *Lax4* specific |
| Lax4-8F | TCTGGTCCAACAAAGCTAGTG | *Lax4* specific |
| Lax4-7R | AGGAATGATGTAGACAGTGAAGG | *Lax4* specific |
| Lax4-8R | ACATGAAATAAAGGGGGAAATTA | *Lax4* specific |
| Lax4-9F | TCCACAAGGATTAAGACAGTGGC | *Lax4* specific |
| Lax4-10F | CAATGTTGTCTCAAAACCAAGC | *Lax4* specific |
| Lax4-11F | TGTGTGGGAGAAGGTTATTGGG | *Lax4* specific |
| Lax4-9R | TGTAGAGTATGTTAGTGGCTC | *Lax4* specific |
| PG3-F | ATATACAAGATACATGCTGTAGATGT |  |
| PG3-R | CCCCTGATGTTCTTGAAGATCACTTT |  |
| PG3-2F | GCAACGCTTGGAATAGCTCAACC |  |
| PG3-3F | AGCTAAGCTCACGGTGAAAA |  |
| PG3-4F | TTGGAGGAGCCTCTAATGCAG |  |
| PG3-2R | TGCTGCAGTTGTTGTGCCCCTG |  |
| PG3-3R | ATGGAGAACTTTGTGTCCACC |  |
| PG11-1R | GGCTTGACATTAGAACATTTAGC | *Pg11c* specific |
| PG11-2R | GCAGGAGATTTTCGGCCAGC | *Pg11c* specific |
| PG11-3R | TTATTTAGCAGGAGATTTTCGGC | *Pg11c* specific |
| PG11-4R | ACATTAGAACATTTAGCTATTGC | *Pg11c* specific |
| PG11-1F | TCATTCCTATTCCTTGCTGAG | *Pg11c* specific |
| PG11-2F | TCCTCCCTAACATTGAAACCA |  |
| PG11-3F | TGCTTGGAAGGAAGCATGTGC |  |
| PG11-4F | CACAGATGGAATCCACATGG |  |
| PG11-5R | GAAACAGTAATTGTTCCTGGTGC |  |
| PG11-6R | AGAAGTAACGCCGGTGACCAT | *Pg11c* specific |
| PG11a1F | CTAAGAACTACTAAAATTACC | *Pg11a* specific |
| PG11a2F | TGATGACATATTCTTATAATTGC | *Pg11a* specific |
| PG11a3F | GTTTCATGCTTGTCCTCTCTCCC | *Pg11a* specific |
| PG11a4F | TGTCAATATAGAGAGTTGACTA | *Pg11a* specific |
| PG11a5F | ATGAAGTTCAGCACTGTCATTA |  |
| PG11a6F | CTTGGAGTGGTAAAGGCGGTAAC |  |
| PG11a1R | CCCAAAACAAGTGGTTTGGCTA | *Pg11a* specific |
| PG11a2R | CAAGGTGTATTTTAGCGTCTTGC | *Pg11a* specific |
| PG11a3R | CTGCAGGAGGAGACCTACGGT | *Pg11a* specific |
| PG11a4R | TCCTCGCATTATGGGGATGGTT |  |
| PG11a5R | TTATTTAGCAGGAGATTTTCCAG |  |
