# Supplementary material for: Contrasted patterns of selective pressure in three recent paralogous gene pairs in the Medicago genus (L.)
Source: BMC Evol Biol. 2012 Oct 1;12:195. doi: 10.1186/1471-2148-12-195 (PMC3517903; doi:10.1186/1471-2148-12-195)

### Additional file 3 – Schematic representation of genes and primers positions

Figure with schematic representation of the intron/exon structure of the 5 sequenced genes on *M. truncatula* (A17) and position of the primers used for the amplification and sequencing.


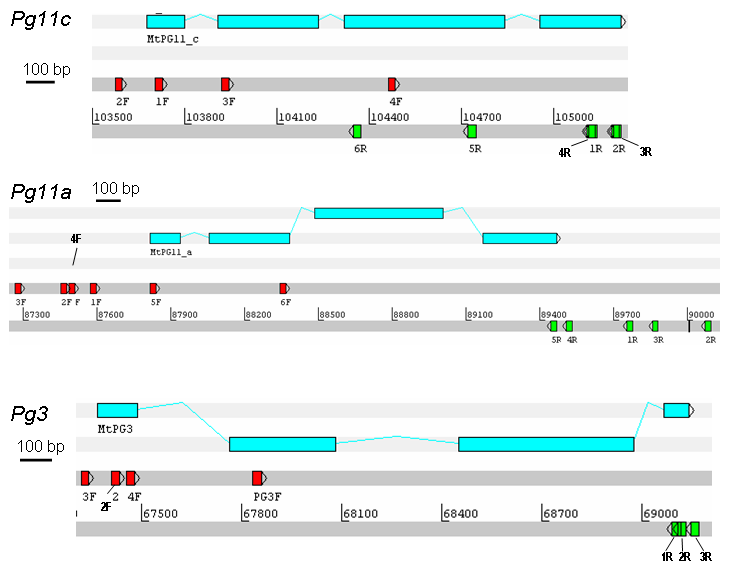


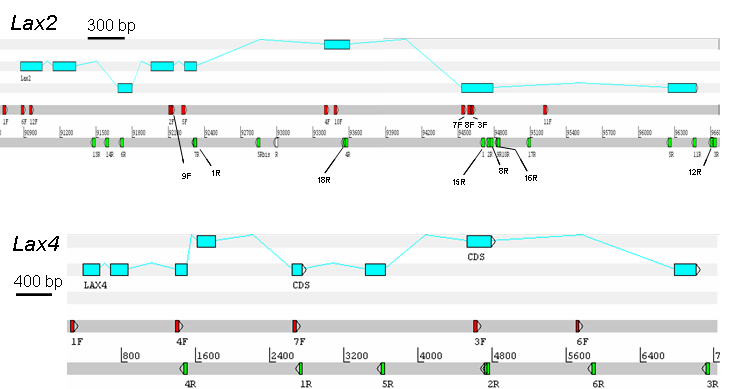

Supplement: Additional file 4 — Schematic representation of genes and primers positions. Figure in PDF format with schematic representation of the intron/exon structure of the 5 sequenced genes on M. truncatula (A17) and position of the primers used for the amplification and sequencing, names and sequences of primers used for amplification and sequencing. [file 1471-2148-12-195-S4.doc]
